# Effect of Electric Current Pulse on Microstructure and Corrosion Resistance of Hypereutectic High Chromium Cast Iron

**DOI:** 10.3390/ma11112220

**Published:** 2018-11-08

**Authors:** Haiyang Lv, Rongfeng Zhou, Lu Li, Haitao Ni, Jiang Zhu, Tong Feng

**Affiliations:** 1 College of Materials and Chemical Engineering, Chongqing University of Arts and Sciences, Chongqing 402160, China; lvhaiyang@cqwu.edu.cn (H.L.); jiangzhu415@cqwu.edu.cn (J.Z.); 2 College of Materials Science and Engineering, Kunming University of Science and Technology, Kunming 650093, China; zhourongfeng@kmust.edu.cn (R.Z.); liluchina@kmust.edu.cn (L.L.); 3 Research Institute for New Materials Technology, Chongqing University of Arts and Sciences, Chongqing 402160, China; fengtong@cqwu.edu.cn

**Keywords:** electric current pulse, high chromium cast iron, primary carbide, morphology, corrosion resistance

## Abstract

The effect of electric current pulse on the microstructure and corrosion resistance of hypereutectic high chromium cast iron was explored. The morphology of carbides in solidification microstructure was observed by an optical microscope and a scanning electron microscope and the composition was determined by an electron probe micro-analyzer. The microhardness of primary carbides and corrosion resistance of samples were also compared. Under the active of electric current pulse, the microstructure of hypereutectic high chromium cast iron was homogenized and its performance improved accordingly. On treatment by electric current, the morphology of primary carbides changed from thick long rods to hexagonal blocks or granular structures. The interlayer spacing of eutectic carbide decreased from ~26.3 μm to ~17.8 μm. Size statistics showed that the average diameter of primary carbide decreased from ~220 μm to ~60 μm. As a result, microhardness increased from 1412 HV to 1511 HV. No obvious microcrack propagation was found at the microindentation sites. The average length of microcracks decreased from ~20.7 μm to ~5.7 μm. Furthermore, corrosion resistance was remarkably enhanced. The average corrosion rate decreased from 2.65 mg/cm^2^·h to 1.74 mg/cm^2^·h after pulse current treatment.

## 1. Introduction

High Chromium Cast Iron (HCCI) containing 11~30 wt% chromium and 2.0~4.3 wt% carbon has been known as the best antifriction material in the world [1,2,3,4]. Due to its good wear resistance and corrosion resistance, HCCI is widely used in building, metallurgy, machinery, chemical industry and other fields [5,6,7,8]. Numerous studies on the relation between microstructure and performance of HCCI have revealed that the more the amount of carbides, the better the wear resistance [9,10]. With regard to the hypereutectic structure, it is usually supported by high content carbide, which has good wear resistance. However, if the morphology of carbides exhibits thick long-rod irregular shape, the toughness of HCCI will decrease, furtherly restricting its industrial application [11]. Based on the reported investigation, if the shape of primary M_7_C_3_ carbide can be converted to fine blocks or granular structures, the wear resistance, corrosion resistance and toughness of HCCI will be comprehensively improved [3,4,10,12,13,14].

In the process treatment of HCCI, pulse current treatment is an effective method to refine carbide without changing the composition of the material. Moreover, this technology offers characteristics of non-pollution to the environment, low cost of preparation and simple operation, attracting a lot of interest in the effect of pulse current treatment on microstructure evolution [15,16,17,18,19,20,21,22]. In the case of hypereutectic HCCI, our previous investigation revealed that obvious refinement effect of primary carbides is observed after pulse current treatment at 45 Hz [23]. However, there is a lack of comparative studies quantitatively investigating the effect of electric current pulse on microstructure and corrosion resistance of hypereutectic HCCI.

In the present investigation, the pulse current is employed during melting and solidification of hypereutectic HCCI. The effect of pulsed current on solidification microstructures and corrosion resistance of hypereutectic HCCI is studied. Particularly, the type, morphology, size and distribution of carbides are focused on.

## 2. Materials and Methods

In order to allocate the required research components, pig iron, high carbon ferrochrome, carbon particles and scrap steel were successively added into 150 kg medium frequency induction furnace for smelting of the hypereutectic HCCI [23]. Keeping the pouring temperature at 1400 °C, the melt was then cast into Φ20 mm × 150 mm cylindrical specimens in a sodium silicate/CO_2_ sand mold. The solidus temperature and liquidus temperature, determined by synchronous differential thermal analyzer (STA 499F3, NETZSCH, Selb, Germany), were 1276 °C and 1337 °C, respectively. The chemical composition is shown in Table 1.

**Figure 1:**
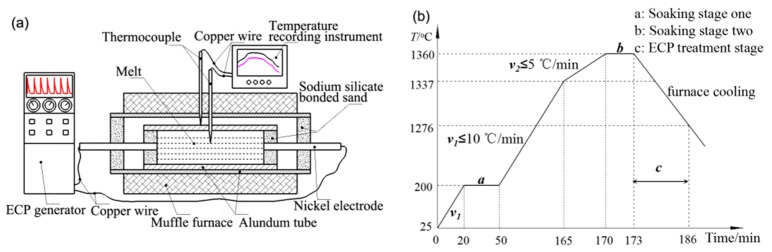
Schematic diagram of ECP treatment device (**a**) and process flow (**b**).

The samples were divided into two groups. Each group of samples were polished and sealed in a tubular resistance furnace (YFK60 × 600/160, Shanghai Yifeng Electric Furnace Co., Ltd., Shanghai, China). The samples were then heated under the established procedures. The experimental device and process curve are shown in Figure 1. When the temperature reached 1360 °C, power was disconnected after 3 min of heat preservation. One of the samples was reconnected to a high voltage pulse power supply (PPCP-5-50-100, Zhonghai Wutong special power supply Co., Ltd., Mianyang, China) for pulsing treatment using vertex sharp waves. The employed pulse voltage, frequency and pulse width were 1200 V, 45 Hz, 10 μs, respectively. When the furnace power was turned off, pulsing treatment was started at the end of soaking stage two and continued until the samples were cooled to 1276 °C. After that, the samples were cooled to room temperature in the furnace. For comparison, the same operating conditions, except pulsing treatment, were employed on another set of samples. During the heating and cooling process, the melt temperature was recorded using TOPRIE-TP700 multi-channel data recorder (Puri Electronics Co., Ltd. of Shenzhen city, Shenzhen, China). For convenience, the sample without electric pulse current treatment was referred to as the non-ECP sample, and the sample with electric pulse current treatment was referred to as the ECP sample.

All specimens were cut radially outwardly from the centers, and subsequently made into small cylindrical specimen with size of Φ15 mm × 20 mm for metallographic preparation. After the samples were etched by 5% FeCl_3_ aqueous solution, the microstructure of the samples was observed by an optical microscope (EZ4D, Leica, Wizlar, Germany). The morphology of the carbides was analyzed by a scanning electron microscope (SEM) (XL30ESEM-TMP, Philips, Amsterdam, The Netherlands). The composition of the primary carbides was determined by electron probe micro-analyzer (JXA8230, JEOL Ltd., Tokyo, Japan). The carbide particles were identified by x-ray diffrcation (D/max-3B, Ricoh Co., Ltd., Tokyo, Japan). Hardness tests were conducted on primary carbides by the microhardness tester (HVS-1000A, Laizhou Weiyi Experiment Machine Co., Ltd., Lanzhou, China). The loading and loading-time were 300 gf and 10 s, respectively.

To investigate the corrosion behavior of the samples, two groups of samples with similar size of Φ10 mm × 4 mm and initial weight of ~24.178 g were cut for corrosion experiment. The samples were completely immersed in excess aqua regia solution (n(HNO_3_):n(HCl) = 1:3) for a 4-stage corrosion. Each stage lasted 21 h. The weight of the sample was measured after each stage. The corresponding corrosion rate was calculated by the weight loss method. Corrosion quantity *A* and corrosion rate *K* of the sample are calculated according to the empirical Formulas (1) and (2).

(1) $$A=(M_{1}-M_{2})/S\;$$

where *M*_1_ and *M*_2_ is the weight before and after corrosion, *S* is the surface area of samples.

(2) K=A/t 

where *t* is corrosion time.

## 3. Results and Discussion

### 3.1. Morphological Properties

**Figure 2:**
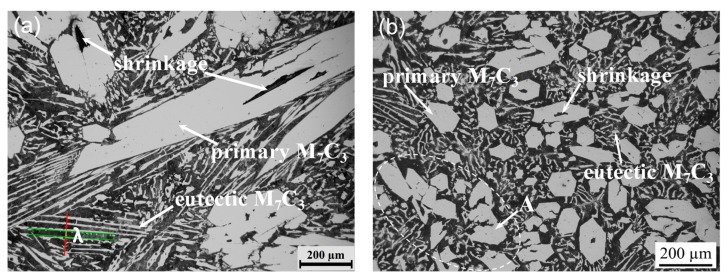
Optical micrographs of solidification microstructure of hypereutectic HCCI with or without ECP treatment: (**a**) Non-ECP treatment; (**b**) ECP treatment.

The typical solidification microstructures of hypereutectic HCCI samples without and with pulse current treatment are shown in Figure 2. Generally, the solidification microstructure of hypereutectic HCCI includes primary M_7_C_3_ carbides, eutectic carbides and matrix [3,4,5,6]. Besides, there are some casting defects such as shrinkage and porosity. For the non-ECP sample, it can be seen from Figure 2a that primary carbides are thick long rod-like, hexagonal or irregular polygon-like structures. Furthermore, the distribution of primary carbides is non-homogeneous in the matrix. The length of some carbides even exceeds 1000 μm. One or more hole defects can be found in most carbides. On the other hand, the eutectic carbides exhibit lamellar agglomeration and are radially distributed. The adjacent eutectic carbide lamellaes have the same orientation [24]. Moreover, quantitative analysis shows that the average lamellar spacing *λ* is ~26.3 μm. However, in the case of the ECP sample, the morphology of primary carbides changes obviously from thick long rod-like to rod-like or polygon block-like structures (Figure 2b). The size of primary carbides decreases significantly and the primary carbides are more dispersed in the matrix. To some extent, a small proportion (~1.3%) of primary carbide is agglomerated, and the holes in the interior become smaller or even disappear. The lamellar spacing *λ* of eutectic carbides is reduced to ~17.8 μm. 

**Figure 3:**
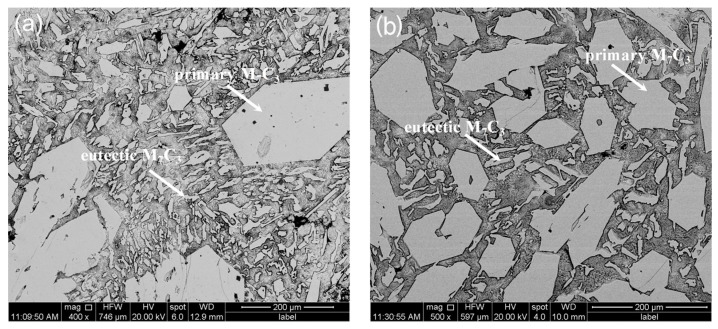
SEM microgrphs of Solidification microstructure of hypereutectic HCCI with or without ECP treatment: (**a**) Non-ECP treatment; (**b**) ECP treatment.

Figure 3 shows SEM micromorphology of the hypereutectic HCCI samples. The microstructurual features captured by SEM is consistent with metallographic observations. Pulse current treatment decreases the size of the primary carbides, which is caused by the inoculation effect of the pulse current in the liquid phase [21]. The pulse current forces the size of the clusters to shrink in the liquid phase. When solidified, these clusters form the core, thus promoting primary carbide nucleation. However, the primary carbide grows via screw dislocation, which results in a great deal of defects [25]. Atoms are easily stacked on the interface step at the defects. Therefore, the crystals exhibit spiral growth and gradually grow into long hexagonal prism-like or rod-like structures [26].

**Figure 4:**
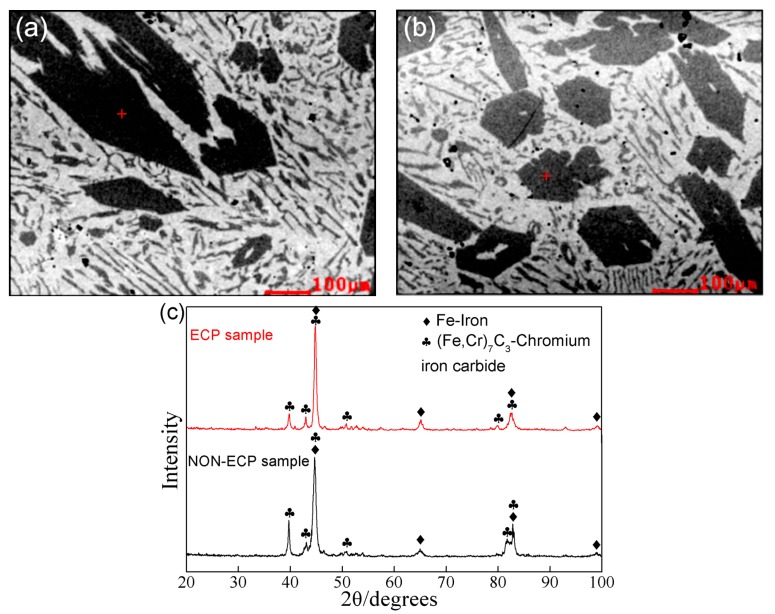
Phase analysis of the primary carbides by EPMA and XRD. The EPMA location on the primary carbides in the (**a**) non-ECP sample and (**b**) ECP sample. (**c**) Typical XRD patterns of the non-ECP sample and ECP sample.

Further phase analysis of the primary carbides was determined by EPMA and XRD. Figure 4a and Figure 4b show the EPMA positions of the non-ECP sample and the ECP sample using SEM images. The average composition of the primary carbides is summarized in Table 2. The results show that the (Fe, Cr)/C ratio of the primary carbides in the two groups of samples agree well with that of the M_7_C_3_ carbides, which is confirmed by phase analysis by XRD (Figure 4c). Thus, it can be seen that pulse current treatment has no effect on the type of primary carbide.

During solidification, according to the magnetic effect of an electric current, a variable magnetic field is produced when the pulse current passes through the metal melt. The electrified melt will be squeezed repeatedly due to the action of magnetic field. In such a process, the melt will lose its superheat rapidly, increase its supercooling degree and influence the diffusion of atoms, resulting in magnetostriction effect [27]. The value of shrinkage stress can be expressed as [27]:

(3) $$\sigma =\nu [\mu j^{2}(r^{2}-a^{2})/4]\;$$

where σ is shrinkage stress, ν is Poisson’s ratio, μ is magnetic conductivity, *j* is current density, *r* is sample radius, *a* is the distance to sample core.

When the primary carbide is nucleated, it will be subjected to shrinkage stress. After nucleation, the grain is compressed repeatedly, and growth is restrained. Therefore, grain size is homogenized and solidified structure is more compact. The greater the current density, the smaller the distance to the sample center, and the greater the shrinkage stress. Due to the inhomogeneity of shrinkage stress, agglomeration of primary carbide can be found (Figure 2b).

**Figure 5:**
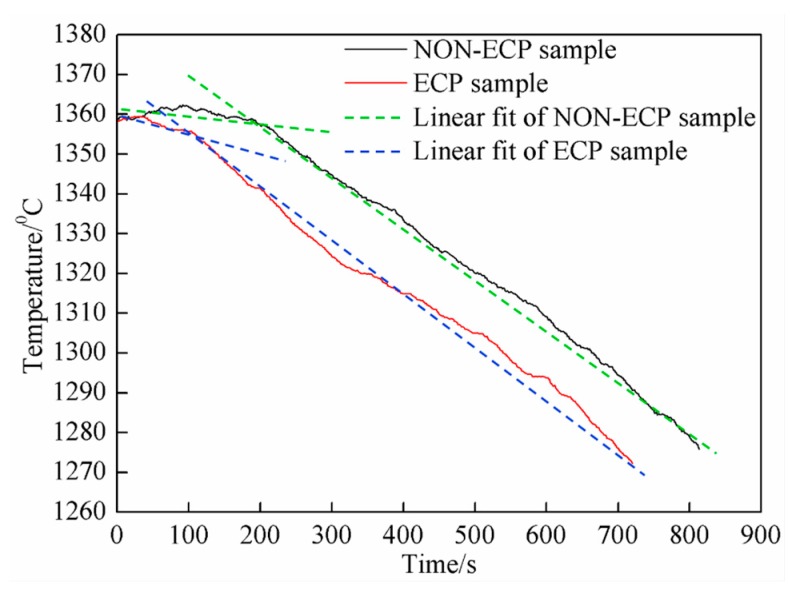
The cooling curve of HHCCI during solidification.

According to data collected by the temperature recorder, the cooling curves of the two groups of samples are drawn in Figure 5. Obviously, the cooling curve can be divided into two stages. In the early stage, it can be seen that the cooling rate of ECP samples is larger than that of the non-ECP samples. Quantitative results show that the cooling rate of non-ECP and ECP samples is 3.7 °C/min and 6.2 °C/min, respectively. However, in the late stage, the cooling rate of non-ECP sample (7.8 °C/min) is basically equal to that of ECP samples (8.2 °C/min). In the furnace-cooling process, the heat dissipation is slow, and the melt temperature does not decrease immediately. Instead, there is an insulation extension process (about 3 min) before slowly decreasing (Figure 5). During solidification, due to slow cooling rate, energy fluctuation and latent heat release, primary carbide crystals can grow continuously, forming long hexagonal prism-like or long rod-like structures (Figure 2a). However, when the pulse current is applied, the magnetic shrinkage effect is produced, making the melt lose its overheating. The prolong process of heat preservation disappears and the temperature decreases obviously. Due to the differences in current density and magnetic field in different parts of the melt, a pressure gradient is produced, and a flow velocity difference is formed locally, which promotes grain refinement [28]. Furthermore, the temperature gradient at the front of the solid-liquid interface increases, which promotes nucleation and increases the nucleation rate. The growth of primary carbide nuclei is inhibited and the preferred orientation is destroyed [29]. Therefore, primary carbides exhibit short rod-like, polygon or hexagonal block-like structures (Figure 2b), and are distributed uniformly in the matrix.

Effect of pulse current on undercooling degree of melt can be given by [30]:

(4) $$\Delta T=kJ_{0}^{2}\xi \pi r^{2}\Delta VT_{m}c^{-1}\;$$

where ΔT is the undercooling degree of melt, *k* is a constant value related to spherical coordinates, $J_{0}$ is the current density before nucleation, $\xi =(\sigma_{1}-\sigma_{2})/(\sigma_{1}+2\sigma_{2})$, $\sigma_{1}$ is the conductivity of crystal nucleus, $\sigma_{2}$ is the conductivity of parent phase, r is sphere radius, ΔV is nucleation volume, $T_{m}$ is melting temperature, ***c*** is mass heat capacity.

Following Equation (4), under the action of a certain current density, when $J_{0}$ is equal to ${\left(\Delta G/k\xi \pi r^{2}\Delta V\right)}^{1/2}$, where ξ>0 and ΔG is maximum nucleation free-energy, the melt can be directly crystallized without supercooling [31]. That is to say, as long as the metal is cooled, it will nucleate, which is equivalent to increasing the undercooling of the melt. With the increase of supercooling degree, the nucleation rate increases. As a result, more embryos are formed, resulting in the formation of a fine-grained structure, and hence the primary carbides are more dispersed. Eutectic carbides are also more dispersed for the same reason.

In particular, the average diameters and microhardness values of primary (Cr, Fe)_7_C_3_ carbide in the two groups of samples are also determined. For non-ECP sample, the average diameter of primary carbide is ~220 μm, and the microhardness value is 1412 HV. For ECP sample, the average diameter of primary carbide is decreased to ~60 μm. Accordingly, the microhardness is increased to 1511 HV.

**Figure 6:**
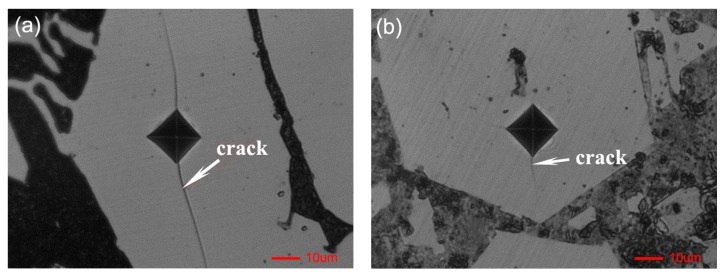
The micro-indentation on the surface of primary carbides: (**a**) Non-ECP sample; (**b**) ECP sample.

The micro indentation on the surface of primary carbides is shown in Figure 6. For non-ECP sample, there are obvious cracks on the surface of the primary carbide, spreading outward along the diagonal line of the microhardness imprint (Figure 6a). The average length of microcracks is ~20.7 μm. However, no obvious crack propagation is found in the ECP sample (Figure 6b). The average length of microcracks is ~5.7 μm, which is much smaller than that of primary carbides in non-ECP samples. These results indicate that the microhardness and fracture toughness of primary carbide as well as the compactness of matrix structure are improved after pulse current treatment.

### 3.2. Corrosion Resistance

**Figure 7:**
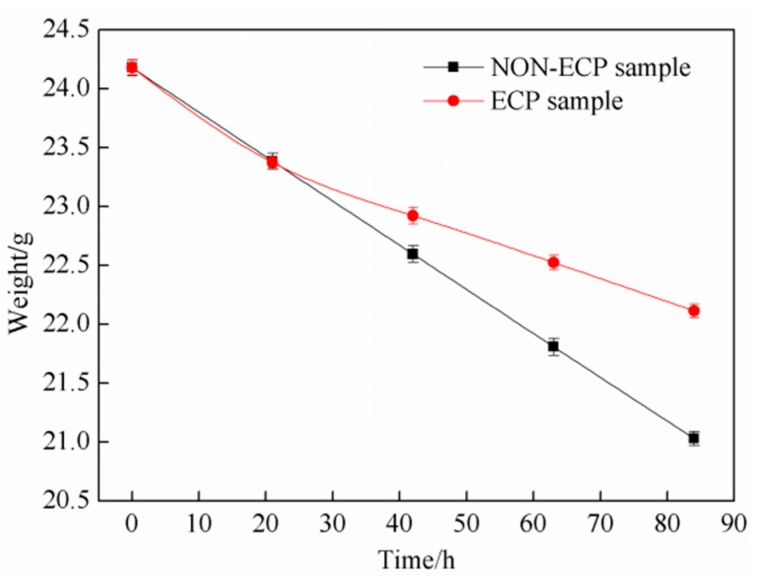
Relationship between weight and corroding time.

The relationship between corrosion weight and corrosion time is shown in Figure 7. With the extension of corrosion time, the weights of the two groups of samples decrease; it is more significant for the non-ECP samples. After 84 h, the weight of the ECP sample was 22.114 g and weight loss was 2.064 g; the weight of the non-ECP sample was 21.029 g and weight loss was 3.149 g.

**Figure 8:**
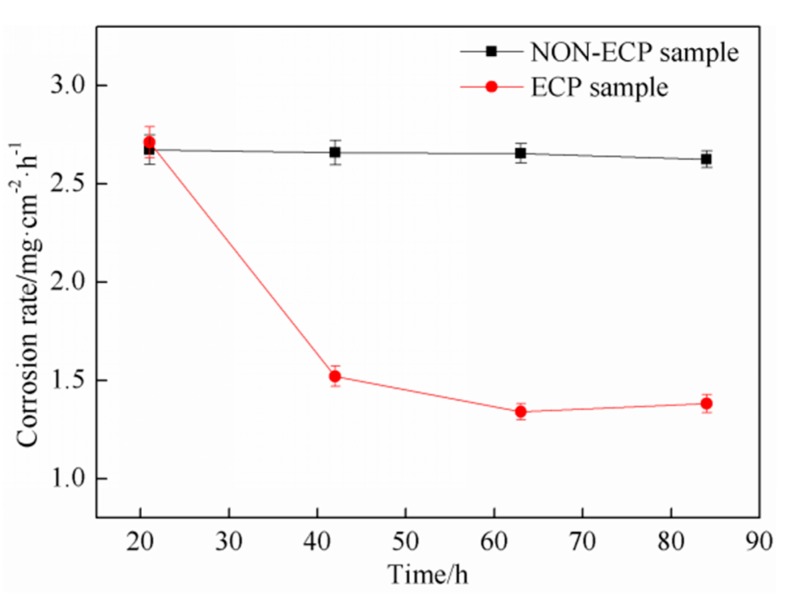
Relationship between corrosion rate and corroding time.

The corrosion rate of the two groups of samples is shown in Figure 8. It is found that the corrosion rate of the non-ECP specimens is basically stable with the increasing corrosion time and the average corrosion rate is 2.65 mg/cm^2^·h. The corrosion rate of ECP samples decreases first and then stays stable; the average value is 1.74 mg/cm^2^·h.

In the first stage of corrosion, the corrosion rates of the two groups of samples are basically the same, indicating that the matrix structure participates in the reaction due to the existence of grain boundaries or defects in the microstructure, and thus corrosion proceeds at a certain rate in the initial stage. However, when the corrosion time exceeds this range, the corrosion degree is obviously different. The weight of non-ECP specimens decreases significantly and the corrosion rate is stable, while the weight of ECP specimens decreases gently and the corrosion rate decreases significantly. This phenomenon suggests that the solid solution Cr element in the matrix increases due to an inoculation effect by pulse current; the potential difference between carbides and matrix decreases and the reaction slows down gradually. Besides, the magnetic contraction effect can promote grain refinement and defect reduction. Therefore, the corrosion rate decreases and the corrosion resistance of the samples increases with increasing time.

**Figure 9:**
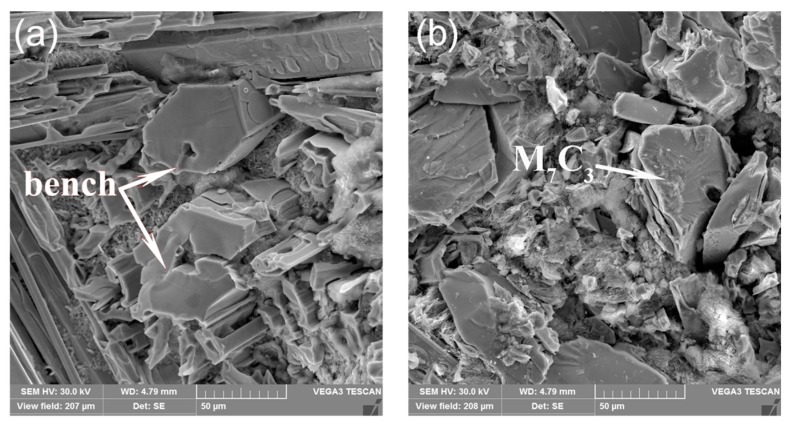

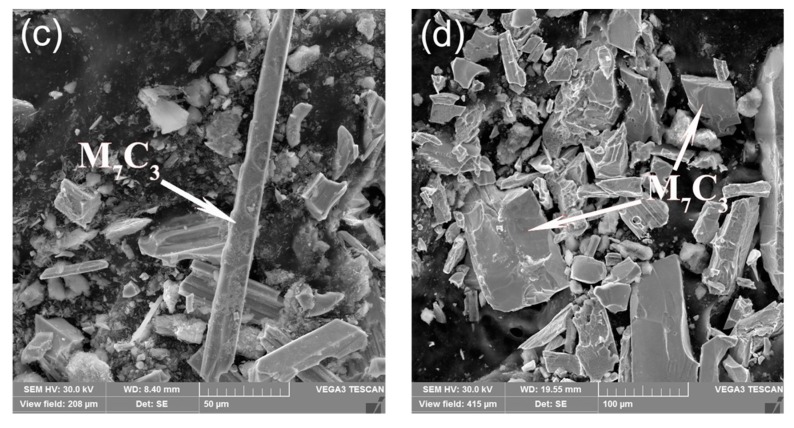
SEM microgrphs of the surface of samples and primary carbides: (**a**) surface of Non-ECP sample; (**b**) surface of ECP sample; (**c**) surface of non-ECP primary carbides; (**d**) surface of ECP primary carbides.

Figure 9 shows the SEM morphology of the surface and primary carbides in the two groups of specimens after deep damage of corrosion. As shown in Figure 9a, the surface of the non-ECP specimens is seriously depressed. The matrix is corroded, and the primary carbide and eutectic carbide are protruded. The end face of the primary carbide is hexagonal, with holes in it. After the impulse current treatment, the corrosion degree of the ECP sample is smaller than that of the non-ECP sample. Although the matrix is corroded, there is no obvious depression on the surface. The primary carbide and eutectic carbide are surrounded by eutectic colonies and there is no bulging phenomenon. The surface of primary carbide is smooth, without obvious defects, holes and spiral steps. By comparison of Figure 9c,d, pulse current treatment can change the morphology of primary carbide from thick rod-like to block-like or granular-like structures; as a result, the size of primary carbide decreases and the microstructure becomes uniform.

## 4. Conclusions

The hypereutectic high chromium iron melt during solidification from 1360 °C to 1276 °C was subjected to a pulse current treatment using 1200 V, 45 Hz and 10 μs pulse width. It is observed that the primary carbide in solidification microstructure is homogenized and its morphology and distribution are improved under the action of pulse current. After pulse current treatment, the morphology of primary carbide changes from thick rod-like to hexagonal block-like or granular-like. Quantitative analysis shows that the average diameter of primary carbides decreases from ~220 μm to ~60 μm, and the microhardness increases from 1412 HV to 1511 HV. The micro indentation result shows that the average length of microcracks decreases from ~20.7 μm to ~5.7 μm. No obvious microcracks expand can be found at the microindentation sites. The interlayer spacing of eutectic carbides decreased from ~26.3 μm to ~17.8 μm. Furthermore, the corrosion resistance of such microstructure is also increased. The average corrosion rate decreases from 2.65 mg/cm^2^·h to 1.74 mg/cm^2^·h. Based on these results for small samples and combined with the current theoretical analysis, it is possible to use pulse current treatment for improving the microstructure and mechanical properties of high chromium iron casts with larger dimensions.

## Figures and Tables

**Table 1:** Chemical composition of hypereutectic high chromium cast iron (wt%).

Element	C	Cr	Si	Mn	Ni	Fe
Content	3.94	20.30	2.10	0.60	0.30	72.76

**Table 2:** Composition of the primary carbides in hypereutectic high chromium cast iron.

Element	C (at%)	Cr (at%)	Fe (at%)	C (wt%)	Cr (wt%)	Fe (wt%)
non-ECP sample	31.27	40.22	28.51	7.17	52.44	40.39
ECP sample	30.39	38.03	31.58	7.45	49.63	42.92
